# Sex differences in factors associated with heart failure and diastolic left ventricular dysfunction: a cross-sectional population-based study

**DOI:** 10.1186/s12889-021-10442-3

**Published:** 2021-02-27

**Authors:** Giulia Cesaroni, Gian Francesco Mureddu, Nera Agabiti, Flavia Mayer, Massimo Stafoggia, Francesco Forastiere, Roberto Latini, Serge Masson, Marina Davoli, Alessandro Boccanelli, on behalf of the PREDICTOR Study Group, A. Boccanelli, G. Cacciatore, G. F. Mureddu, V. Rizzello, N. Agabiti, G. Cesaroni, F. Forastiere, C. A. Perucci, M. Davoli, F. Colivicchi, M. Santini, R. Latini, S. Masson, M. Uguccioni, M. Iacomelli, M. Di Gennaro, F. Qualandri, V. Paniccia, F. Ammirati, R. Donati, R. Fiaschetti, G. Barbato, T. A. Gaspardone, G. Vitaliani, F. Catalano, A. Achilli

**Affiliations:** 1 Department of Epidemiology - Regional Health Service, ASL Roma 1, Via C. Colombo 112, 00147 Rome, Italy; 2 Cardiology and Cardiovascular Rehabilitation Unit, S. Giovanni-Addolorata Hospital, Rome, Italy; 3 National Center for Disease Prevention and Health Promotion, National Institute of Health, Rome, Italy; 4 Mario Negri Institute for Pharmacological Research – IRCCS, Milan, Italy; 5 Quisisana Clinic, Rome, Italy

**Keywords:** Heart failure, Diastolic left ventricular dysfunction, Sex differences, Risk factors, Elderly, NT-proBNP

## Abstract

**Background:**

Although sex differences in cardiovascular diseases are recognised, including differences in incidence, clinical presentation, response to treatments, and outcomes, most of the practice guidelines are not sex-specific. Heart failure (HF) is a major public health challenge, with high health care expenditures, high prevalence, and poor clinical outcomes. The objective was to analyse the sex-specific association of socio-demographics, life-style factors and health characteristics with the prevalence of HF and diastolic left ventricular dysfunction (DLVD) in a cross-sectional population-based study.

**Methods:**

A random sample of 2001 65–84 year-olds underwent physical examination, laboratory measurements, including N-terminal pro-B-type natriuretic peptide (NT-proBNP), electrocardiography, and echocardiography. We selected the subjects with no missing values in covariates and echocardiographic parameters and performed a complete case analysis. Sex-specific multivariable logistic regression models were used to identify the factors associated with the prevalence of the diseases, multinomial logistic regression was used to investigate the factors associated to asymptomatic and symptomatic LVD, and spline curves to display the relationship between the conditions and both age and NT-proBNP.

**Results:**

In 857 men included, there were 66 cases of HF and 408 cases of DLVD (77% not reporting symptoms). In 819 women, there were 51 cases of HF and 382 of DLVD (79% not reporting symptoms). In men, the factors associated with prevalence of HF were age, ischemic heart disease (IHD), and suffering from three or more comorbid conditions. In women, the factors associated with HF were age, lifestyles (smoking and alcohol), BMI, hypertension, and atrial fibrillation. Age and diabetes were associated to asymptomatic DLVD in both genders. NT-proBNP levels were more strongly associated with HF in men than in women.

**Conclusions:**

There were sex differences in the factors associated with HF. The results suggest that prevention policies should consider the sex-specific impact on cardiac function of modifiable cardiovascular risk factors.

**Supplementary Information:**

The online version contains supplementary material available at 10.1186/s12889-021-10442-3.

## Background

Heart failure (HF) is a clinical syndrome characterised by symptoms and signs of increased tissue/organ fluid retention and decreased tissue/organ perfusion [1–4]. Together with the ageing of the population, the prevalence of heart failure continues to increase worldwide, and it has high rates of morbidity and mortality, leading to enormous human, social, and economic costs [4]. Thus, the growing epidemic of heart failure is one of the major health problems in the developed countries [4]. Since people with heart failure develop symptoms gradually, given the progressive nature of the disease characterised by a long preclinical phase, early interventions to prevent the disease are hypothetically possible [1–4]. Early recognition of clinical HF is critical to prevent recurrences of HF and hospitalisations due to decompensation [1].

Sex differences in the prevalence, presentation, management, and outcomes of different cardiovascular diseases have been found, and gender-specific medicine has received growing attention in recent years [5–9]. Sex differences in the presentation of HF may play an important role in the progression of the disease, in the development of relevant prognostic comorbidities, and even in the response to therapies [10, 11].

Although sex is recognised as a modifier of health, disease, and medicine, the diagnostic and therapeutic approaches are not differential by sex [12, 13]. The present study aimed at evaluating the independent association of traditional cardiovascular risk factors with HF and diastolic left ventricular dysfunction (DLVD) in men and women aged 65–84 years from the PREDICTOR study database. In particular, we investigated whether there are sex differences in the association between age and the prevalence of the diseases, and whether there are sex differences in the association of N-terminal pro-B-type natriuretic peptide (NT-proBNP) and the prevalence of HF and DLVD.

## Methods

### The PREDICTOR study

PREDICTOR is a cross-sectional population-based study. The design, study population, and procedures have been described elsewhere [14, 15]. Briefly, a random sample of 5940 residents, aged 65–84, from four cities in the Lazio region (5.5 million inhabitants) was identified using the Regional Health Registry of 1 June 2007. The final sample size was determined a priori to estimate a prevalence of 3% for HF and of 30% for LVD with a significance level of P 0.05 assuming a 30% participation rate. In total, 5940 people were invited to participate by mail and were informed of the aims and the methodology of the survey. The reasons for refusal to participate included old age, major disability, severe comorbidities, and difficulties in reaching the cardiology center.

In total, 2001 subjects agreed to participate and underwent physical examination, electrocardiography, and echocardiography at eight cardiology centres.

Sociodemographic variables (age, sex, and educational level), lifestyle factors (smoking, alcohol consumption, physical activity) and health characteristics were recorded in a case report form by physicians or trained nurses at eight cardiology centres [14].

As previously reported [14], colour Doppler echocardiograms were performed in peripheral centres using a predefined standard protocol, were recorded with standard DICOM format, centrally analysed by two independent observers, and reviewed by one experienced reader. The two dimensional (2D) parasternal long axis view or the M-mode parasternal short axis recording were used to obtain linear measurements of cardiac chambers. Linear measurements of the left ventricle were used to determine the left ventricle volumes, by using prognostic validated formulas [14]. When linear measures of the LV were not available, LV volumes were obtained from the apical four-chamber view and the EF was calculated using the modified Simpson’s rule method. Diastolic LVD was defined using doppler-derived indexes of transmitral flow and pulmonary vein flow, and tissue Doppler imaging of the lateral mitral annulus (E/e’) according with current guidelines.

The assessment of anthropometric measures, blood pressure, and heart rate was conducted following MONICA recommendations. Information about clinical history and comorbidities was retrieved for each individual: dyslipidaemia, diabetes mellitus, hypertension, family history of cardiovascular diseases, ischemic heart disease (including acute myocardial infarction, angina pectoris, and revascularisation procedures), atrial fibrillation, other cardiovascular diseases (including peripheral vascular and valve disease), and chronic comorbid conditions (including cerebrovascular diseases, chronic obstructive pulmonary disease, liver disease, thyroid disorders, blood diseases, gastric disorders, renal disease, Parkinson’s disease, other diseases of the central nervous system, and cancer) [14]. Moreover, information on medications (including diuretics and angiotensin II receptor blockers, ARBs, mineralocorticoid receptor antagonists, beta blockers, statins, antiarrhythmic drugs, antiaggregant drugs, and calcium channel blockers) continuously used in the 6 months before the visit were collected.

As previously reported, fasting blood samples were collected and standard laboratory tests were performed locally. N-terminal pro brain natriuretic peptide (NT-proBNP) was blindly measured, with an electrochemiluminescence immunoassay (Elecsys 2010, Roche Diagnostics GmbH) in a central laboratory [14].

### Conditions under study

The diagnosis of HF was based on the clinical evaluation (clinical history, plus symptoms and signs of HF) done in peripheral centres according to the 2005 European Society of Cardiology (ESC) criteria as previously reported. Then, subjects with a clinical diagnosis of HF and in New York Heart Association (NYHA) class 1 were checked centrally, and the diagnosis of HF was validated only when all the three conditions (clinical diagnosis, NYHA class 1, and LVD confirmed by the central echo lab) occurred.

Diastolic function was defined as normal if at least three of the following conditions were satisfied: E/A ratio > 0.75 and < 1.5; DTE > 140 ms and < 280 ms; PV peak S > PV peak D, PVa dur-Adur difference < 0, and E/e’ < 8, based on a specific algorithm that took into account the existing recommendations and guidelines [14]. When fewer than three diagnostic criteria were recognized in the case of discordant parameters, or when an equal number of criteria were recognized for more than one category of diastolic function, DLVD was defined as indeterminate.

In the current study, we investigated HF, DLVD, and DLVD in the presence or absence of reported symptoms such as fatigue, dyspnoea, oedema, tachycardia, or any limitation in daily activities (symptomatic and asymptomatic DLVD).

### Statistical analysis

We selected all subjects with no missing values in sociodemographic variables, health characteristics, NT-proBNP measurements, and echocardiographic variables. The sociodemographic and health characteristics of the participants and subjects affected by HF and DLVD were tabulated by sex. Similarly, the characteristics were tabulated by absence of DLVD, presence of asymptomatic DLVD, and presence of symptomatic DLVD. We used Χ^2^ to compare the distributions of the categorical variables in the men and women as in the different categories of DLVD, a t-test to compare the means of the continuous variables by sex, and linear regression analyses to compare continuous variables across the categories of DLVD.

To analyse the relationship between age and the conditions under study in men and women, we modelled age as a cubic spline using a generalised additive model [16]. We used the likelihood ratio test to compare the model with age as a natural spline vs. the model with age as a linear term. Similarly, we plotted cubic splines to investigate the shape of the relationship between the logarithmic transformations of NT-proBNP measurements and the conditions.

We used multivariable logistic regression models to evaluate the association (odds ratios and 95% confidence intervals, OR and 95% CI) between the investigated factors and the conditions under study separately for men and women. We performed age-adjusted and fully-adjusted analyses. We used the likelihood ratio test to investigate the possible interaction between the factors and sex.

To analyse the characteristics of subjects with asymptomatic and symptomatic DLVD compared to those without a diagnosis of DLVD we performed age-adjusted and fully-adjusted multinomial logistic regression models, using as outcome a categorical variable indicating the absence of DLVD, the presence of asymptomatic DLVD, the presence of symptomatic DLVD.

Since we used a complete case analysis approach, we excluded from our analysis 325 records (16% of the original 2001 sample). To investigate whether the selection of all subjects with complete data might have introduced a bias, we used an inverse probability approach [17, 18]. A proportion of exclusion was due to missing sociodemographic and health variables, 64% of exclusion was due to missing echocardiographic parameters. Through a multivariable logistic regression, we estimated the probability of being included and weighted the included population taking account of the characteristics of those not included. We used age, sex, IHD, other cardiovascular diseases and other comorbid conditions to calculate a weight [18]. We then replicated the analyses using the weights.

## Results

Table 1 describes the demographic, clinical, and behavioural characteristics in men and women included in the study, and in the subgroups of individuals affected by HF and DLVD. Compared to women, men were highly educated, had a higher proportion of smoking and alcohol consumption, were more active, had a different distribution of BMI categories (with a higher proportion of overweight), had a lower prevalence of dyslipidaemia, and of family history of cardiovascular disease, but higher prevalence of diabetes and ischemic heart disease (IHD). In the entire population, creatinine level was higher in men than in women, the % ejection fraction was lower in men than in women, and NT-proBNP was lower in men than in women. Overall, men used more than women angiotensin converting enzyme inhibitors, calcium channel blockers, and antiaggregant drugs.

**Table 1:** Distribution (%) of socio-demographic, lifestyle, and health characteristics in the study population and in subjects suffering from heart failure (HF) and diastolic left ventricular dysfunction (DLVD) by gender

Characteristic		Men			Women		
		Total ***N*** = 857	HF ***N*** = 66	DLVD ***N*** = 408	Total ***N*** = 819	HF ***N*** = 51	DLVD ***N*** = 382
**Age** (m, sd)		73 (5)	76 (5)	74 (5)	73 (5)	75 (5)	74 (5)
**Education level**	<= Primary School	27.0*	37.9	27.7*	39.4*	43.1	42.4*
	Junior High School	22.1*	21.2	19.9*	22.6*	25.5	21.7*
	> = High School	50.9*	40.9	52.4*	38.0*	31.4	35.9*
**Smoking habit**	Never	30.5*	15.1*	28.4*	66.5*	56.9*	67.0*
	Ever	69.5*	84.9*	71.6*	33.5*	43.1*	33.0*
**Alcohol consumption**	No	26.8*	36.4	27.2*	56.2*	47.1	55.8*
	Yes	73.2*	63.6	72.8*	43.8*	52.9	44.2*
**Physical activity**	No	52.9*	57.6	56.1*	62.7*	76.5	64.7*
	Occasionally	23.4*	24.2	21.1*	20.0*	11.8	18.8*
	Daily	23.7*	18.2	22.8*	17.3*	11.8	16.5*
**BMI**	> = 30	14.8*	18.2	16.2*	17.7*	31.4	20.4*
	25–29.9	51.2*	54.6	51.5*	35.3*	41.2	37.4*
	< 25	34.0*	27.3	32.3*	47.0*	27.5	42.2*
**Dyslipidemia**	No	63.4*	72.7*	65.4*	47.7*	45.1*	50.3*
	Yes	36.6*	27.3*	34.6*	52.3*	54.9*	49.7*
**Diabetes**	No	80.9*	74.2	76.2*	86.4*	86.3	83.5*
	Yes	19.1*	25.8	23.8*	13.6*	13.7	16.5*
**Hypertension**	No	41.7	25.8	37.0	40.8	17.7	36.7
	Yes	58.3	74.2	63.0	59.2	82.4	63.4
**Family history of CVD**	No	82.5*	75.8	83.6*	75.0*	64.7	73.8*
	Yes	17.5*	24.2	16.4*	25.0*	35.3	26.2*
**Ischemic heart disease**^**a**^	No	82.6*	54.5*	78.2*	92.4*	86.3*	91.9*
	Yes	17.4*	45.5*	21.8*	7.6*	13.7*	8.1*
**Atrial fibrillation**	No	92.3	81.8	90.4	93.4	84.3	92.9
	Yes	7.7	18.2	9.6	6.6	15.7	7.1
**Other cardiovascular disease**^**b**^	No	94.0	89.4	93.4	93.9	94.1	93.5
	Yes	6.0	10.6	6.6	6.1	5.9	6.5
**Other conditions**^**c**^	No	52.4*	40.9	52.2*	43.0*	35.3	43.7*
	1–2	40.6*	45.5	38.7*	46.7*	56.9	47.9*
	3+	7.0*	13.6	9.1*	10.3*	7.8	8.4*
**Use of diuretics**	No	77.7*	53.0	73.8	73.1*	39.2	68.9
	Yes	22.3*	47.0	26.2	26.9*	60.8	31.1
**Use of ARBs**	No	77.0	74.2*	75.7	75.2	56.9*	72.0
	Yes	23.0	25.8*	24.3	24.8	43.1*	28.0
**Use of Angiotensin-converting enzyme inhibitors**	No	71.3*	53.0	67.4*	78.7*	70.6	76.7*
	Yes	28.7*	47.0	32.6*	21.3*	29.4	23.3*
**Mineralocorticoid receptor antagonists**	No	98.1	92.4	97.8	98.9	94.1	98.7
	Yes	1.9	7.6	2.2	1.1	5.9	1.3
**Beta blockers**	No	82.2	68.2	78.9	82.3	68.6	81.2
	Yes	17.8	31.8	21.1	17.7	31.4	18.8
**Statins**	No	76.8	66.7	76.0	73.6	66.7	75.4
	Yes	23.2	33.3	24.00	26.4	33.3	24.6
**Antiarrhythmic drugs**	No	96.0	90.9	95.1	96.2	92.2	96.3
	Yes	4.0	9.1	4.9	3.8	7.8	3.7
**Antiaggregant drugs**	No	68.7*	56.1	64.9*	74.9*	56.9	73.0*
	Yes	31.3*	43.9	35.1*	25.2*	43.1	27.0*
**Calcium channel blockers**	No	79.1*	75.8	77.0	83.6*	66.7	82.2
	Yes	20.9*	24.2	23.0	16.4*	33.3	17.8
**Creatinine level (mg/dl) (mean, sd)**		1.06 (0.27)*	1.11 (0.30)*	1.09 (0.30)*	0.85 (0.24)*	0.90 (0.30)*	0.87 (0.23)*
**% Ejection Fraction (mean,sd)**		65.0 (8.1)*	53.0 (12.6)*	62.5 (9.6)*	67.4 (6.3)*	62.7 (11.8)*	66.4 (6.9)*
**E/A wave ratio**		0.81 (0.27)	0.84 (0.53)	0.77 (0.34)	0.80 (0.23)	0.79 (0.43)	0.73 (0.25)
**E/e’**		7.7 (2.8)*	9.5 (3.1)*	8.9 (2.9)*	8.6 (3.4)*	11.2 (4.4)*	9.9 (3.9)*
**NT-pro BNP (pg/mL)(median, P25-P75)**		78 (40–174)*	389 (119–1117)*	107 (49–249)*	102 (53–180)*	129 (76–399)*	105 (56–185)*

^a^ Either angina pectoris, or myocardial infarction, or revascularization procedures

^b^ Either peripheral vascular disease or valve disease

^c^ Number of other conditions among stroke, TIA, COPD, liver disease, thyroid disorders, blood disease, gastric disorders, renal disease, Parkinson’s disease, other disease of CNS, and cancer

* The difference between men and women in the total population or between men and women in the subgroups of individuals affected by HF and DLVD was statistically significant with *p* < 0.05

In heart failure subjects, the different distribution of risk factors by sex was attenuated in comparison to the general population. Still, compared to women, men with HF were more frequently smokers, less dyslipidaemic, more frequently had an history of IHD, had a higher NT-proBNP, and used less ARBs. A total of 408 men (48%) and 47% of women had a diagnosis of DLVD. The men and women with DLVD had characteristics similar to the overall population. The majority (77 and 79%, respectively) of subjects with DLVD were asymptomatic (ADLVD): they did not report symptoms such as fatigue, dyspnoea, oedema, tachycardia, or any limitation in daily activities.

**Fig. 1:**
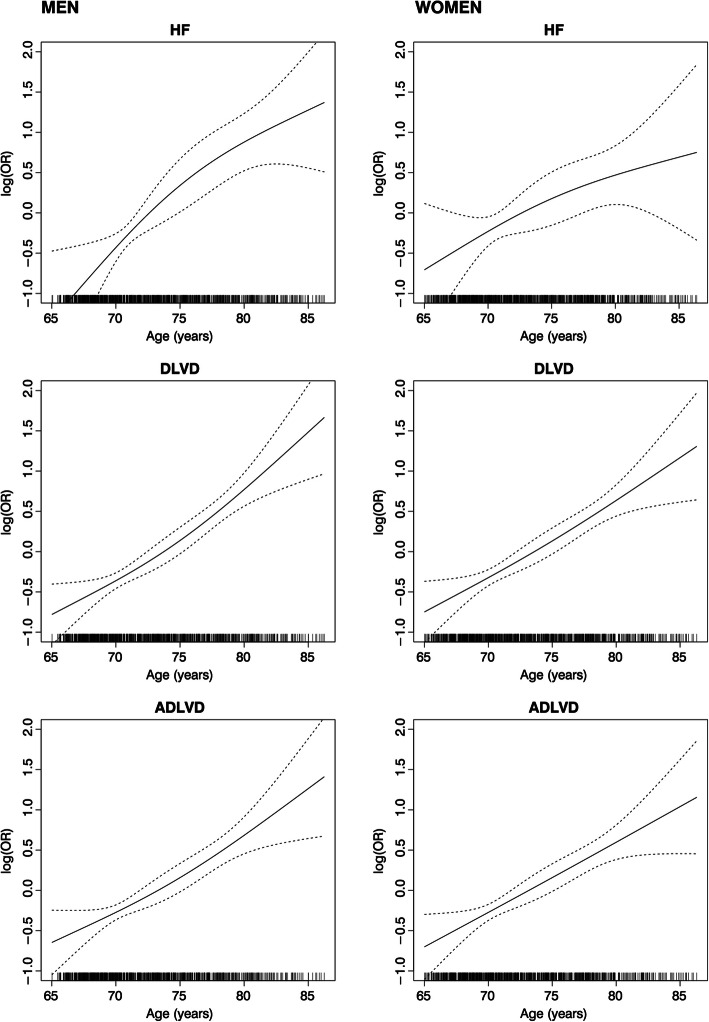
Relationship between age and heart failure (HF), diastolic left ventricular dysfunction (DLVD), and asymptomatic DLVD (ADLVD) in men and women

Age was an important determinant of all the conditions considered (*p* < 0.001). Figure 1 reports the relationship between age (as a non-linear term) and the logarithm OR of the conditions under study separately for men and women. The likelihood ratio test suggests no better fit of the spline model compared to the linear one in both sexes (*p* > 0.08), with the exception of the relationship between age and HF in the men. Although there was no evidence of effect modification in the relationship between age and HF by sex, the slope of the curve was steeper in men than in women with HF: for each year of increasing age, an OR = 1.15 (95% CI: 1.09–1.20) in men vs OR = 1.08 (95% CI: 1.02–1.14) in women. The curves of the association between age and DLVD (overall and asymptomatic) were similar in the two sexes.

Supplemental Figure 1 shows the curves of the crude relationship between the logarithm of NT-proBNP measurements and the conditions in both sexes. The relationship was linear and steep in men for both the conditions, whereas it was U-shaped for women.

Table 2 shows the distribution of socio-demographic, lifestyle and health characteristics in men and women by DLVD category: absence of diastolic left ventricular dysfunction, presence of DLVD without symptoms, and presence of DLVD with symptoms. The differences between men and women confirm the differences presented in Table 1. In men, across categories of DLVD, there were differences in age, smoking habit, diabetes, hypertension, ischemic heart disease, atrial fibrillation, other comorbid conditions, and in use of beta blockers, antiarrhythmic and antiaggregant drugs. In women, across categories of DLVD, there were differences in age, BMI, hypertension, and in use of ARBs, and beta blockers. In both genders, the use of diuretics increased with worsening of DLVD, as NTproBNP and creatinine levels. Similarly, echography variables (% ejection fraction, E/A wave ratio, and E/e’) worsened across levels of DLVD in both men and women (with a statistically significant trend).

**Table 2:** Distribution (%) of socio-demographic, lifestyle, and health characteristics in men and women by absence of diastolic left ventricular dysfunction (DLVD), presence of DLVD without symptoms (ADLVD), and presence of DLVD with symptoms (SDLVD)

Characteristic		Men			Women		
		Without DLVD ***N*** = 449	ADLVD ***N*** = 315	SDLVD ***N*** = 93	Without DLVD ***N*** = 437	ADLVD ***N*** = 301	SDLVD ***N*** = 81
**Age** (m, sd) †‡		72 (4)	74 (5)	76 (5)	72 (5)	74 (5)	75 (5)
**Education level**	<= Primary School	26.3*	25.4*	33.5	36.8*	41.9*	44.5
	Junior High School	24*	20.6*	17.2	23.3*	21.6*	22.2
	> = High School	49.7*	54.0*	47.3	39.8*	36.5*	33.3
**Smoking habit** †	Never	32.3*	31.8*	17.2*	66.1*	67.1*	66.7*
	Ever	67.7*	68.2*	82.8*	33.9*	32.9*	33.3*
**Alcohol consumption**	No	26.5*	26.0*	31.2*	56.5*	55.5*	56.8*
	Yes	73.5*	74.0*	68.8*	43.5*	44.5*	43.2*
**Physical activity**	No	49.9*	54.6	61.3	60.9*	62.1	74.1
	Occasionally	25.6*	21.0	21.5	21.1*	20.3	13.6
	Daily	24.5*	24.4	17.2	18.0*	17.6	12.3
**BMI** ‡	> = 30	13.6*	15.9*	17.2	15.3*	18.6*	27.2
	25–29.9	51.0*	50.5*	54.8	33.4*	33.9*	50.6
	< 25	35.4*	33.6*	28.0	51.3*	47.5*	22.2
**Dyslipidemia**	No	61.5*	64.1*	69.9*	45.5*	50.8*	48.2*
	Yes	38.5*	35.9*	30.1*	54.5*	49.2*	51.8*
**Diabetes** †	No	85.1	75.9*	77.4	89.0	83.4*	84.0
	Yes	14.9	24.1*	22.6	11.0	16.6*	16.0
**Hypertension** †‡	No	45.9	39.0	30.1	44.4	39.9	24.7
	Yes	54.1	61.0	69.9	55.6	60.1	75.3
**Family history of CVD**	No	81.5*	85.1*	78.5	76.0*	75.7*	66.7
	Yes	18.5*	14.9*	21.5	24.0*	24.3*	33.3
**Ischemic heart disease**^**a**^ †	No	86.6*	83.8*	59.1*	92.9*	93.0*	87.7*
	Yes	13.4*	16.2*	40.9*	7.1*	7.0*	12.3*
**Atrial fibrillation** †	No	94.0	93.0	81.7	93.8	94.0	88.9
	Yes	6.0	7.0	18.3	6.2	6.0	11.1
**Other cardiovascular disease**^**b**^	No	94.7	94.6	89.3	94.3	94.0	91.4
	Yes	5.3	5.4	10.7	5.7	6.0	8.6
**Other conditions**^**c**^ †	No	52.6*	55.2	41.9*	42.3*	46.2	34.6*
	1–2	42.3*	38.1	40.9*	45.8*	44.5	60.5*
	3+	5.1*	6.7	17.2*	11.9*	9.3	4.9*
**Use of diuretics** †‡	No	81.3	76.5	64.5	76.9	73.8	50.6
	Yes	18.7	23.5	35.5	23.1	26.3	49.4
**Use of ARBs** ‡	No	78.2	75.9	75.3	78.0	74.8	61.7
	Yes	21.8	24.1	24.7	22.0	25.3	38.3
**Use of Angiotensin-converting enzyme inhibitors** †	No	74.8*	70.8	55.9	80.6*	77.1	75.3
	Yes	25.2	29.2	44.1	19.5	22.9	24.7
**Mineralocorticoid receptor antagonists**	No	98.4	98.7	94.6	99.1	99.3	96.3
	Yes	1.6	1.3	5.4	0.9	0.7	3.7
**Beta blockers** †‡	No	85.1	81.9	68.8	83.3	83.7	71.6
	Yes	14.9	18.1	31.2	16.7	16.3	28.4
**Statins**	No	77.5	78.4	67.7	72.1	76.1	72.8
	Yes	22.5	21.6	32.3	27.9	23.9	27.2
**Antiarrhythmic drugs** †	No	96.9	96.8	89.3	96.1	96.7	95.1
	Yes	3.1	3.2	10.8	3.9	3.3	4.9
**Antiaggregant drugs** †	No	72.2	67.3	57.0	76.4	74.4	67.9
	Yes	27.8	32.7	43.0	23.6	25.6	32.1
**Calcium channel blockers**	No	81.1	77.8*	74.2	84.9	84.1*	75.3
	Yes	18.9	22.2*	25.8	15.1	16.0*	24.7
**Creatinine level (mg/dl)** **(mean, sd)** †‡		1.04 (0.23)*	1.08 (0.29)*	1.14 (0.33)*	0.84 (0.24)*	0.86 (0.21)*	0.91 (0.26)*
**% Ejection Fraction (mean,sd)** †‡		67.1 (5.8)*	64.1 (8.1)*	56.9 (12.1)*	68.2 (5.5)*	67.1 (6.2)*	64.0 (8.7)*
**E/A ratio (mean,sd)** †‡		0.85 (0.18)	0.74 (0.25)	0.86 (0.25)	0.86 (0.18)	0.72 (0.21)	0.76 (0.37)
**E/e’ (mean,sd)** †‡		6.7 (2.2)*	8.8 (2.8)*	9.3 (3.2)*	7.4 (2.4)*	9.6 (3.3)*	11 (5.6)*
**NT-pro BNP (pg/mL) (median, P25-P75)** †‡		63 (33–125)*	88 (42–182)	291 (92–756)*	99 (51–177)*	96 (53–167)	132 (78–276)*

^a^Either angina pectoris, or myocardial infarction, or revascularization procedures

^b^Either peripheral vascular disease or valve disease

^c^Number of other conditions among stroke, TIA, COPD, liver disease, thyroid disorders, blood disease, gastric disorders, renal disease, Parkinson’s disease, other disease of CNS, and cancer

*The difference between men and women in the total population or between men and women in each condition is statistically significant *p* < 0.05

†The differences across groups of DLVD are statistically significant in men (*p* < 0.05)

‡The differences across groups of DLVD are statistically significant in women (*p* < 0.05)

3

Table 3 shows the association between sociodemographic and health factors with the presence of HF, separately in men and in women. The results from fully adjusted models (adjusted for all the variables in the table) show that the characteristics associated with HF were age, IHD, and the presence of more than two comorbid conditions in men; whereas they were age, smoking habit, alcohol consumption, hypertension, atrial fibrillation, and BMI in women. When we introduced NT-proBNP in the model, taking account of all the factors in the table, we found a stronger association of NT-proBNP with HF in men compared to women (likelihood ratio test p for interaction = 0.0229). For each increase in the log transformation of NT-proBNP we found an OR = 2.54 (95%CI: 1.88–3.44) in men and an OR = 1.46 (95%CI: 1.04–2.06) in women.

**Table 3:** Association between characteristics of the population and heart failure (HF) in men and women. Adjusted Odds Ratios (ORs) and 95% Confidence Intervals (CI)

	Men						Women					
	Age adjusted OR	95% CI		Fully adjusted OR	95% CI		Age adjusted OR	95% CI		Fully adjusted OR	95% CI	
**Age** (1 year increase)	**1.15**	**1.09**	**1.20**	**1.14**	**1.07**	**1.21**	**1.08**	**1.02**	**1.14**	**1.09**	**1.03**	**1.16**
**Education level**												
Junior High vs. Primary School	0.85	0.42	1.73	1.01	0.48	2.15	1.09	0.53	2.22	1.27	0.59	2.73
> = High vs. Primary School	0.63	0.35	1.14	0.71	0.38	1.33	0.81	0.41	1.58	1.03	0.49	2.18
**Smoking habit** (Ever vs. Never)	**2.31**	**1.15**	**4.64**	2.04	0.97	4.31	1.62	0.91	2.88	**2.02**	**1.08**	**3.79**
**Alcohol consumption** (Yes vs. No)	0.63	0.37	1.08	0.64	0.36	1.13	1.53	0.87	2.70	**1.98**	**1.07**	**3.69**
**Physical activity**												
Occasionally vs. No	1.13	0.60	2.11	1.12	0.57	2.18	0.51	0.21	1.23	0.49	0.19	1.23
Daily vs. No	0.74	0.37	1.47	0.76	0.37	1.56	0.58	0.24	1.41	0.54	0.21	1.39
**BMI**												
25–29.9 vs. < 25	1.35	0.75	2.44	1.93	0.82	4.50	**3.29**	**1.56**	**6.92**	**3.47**	**1.52**	**7.92**
30+ vs. < 25	1.58	0.74	3.39	1.49	0.78	2.84	**2.08**	**1.04**	**4.16**	**2.12**	**1.01**	**4.45**
**Dyslipidemia** (Yes vs. No)	0.72	0.41	1.28	0.47	0.25	0.90	1.07	0.61	1.88	1.02	0.55	1.90
**Diabetes** (Yes vs. No)	1.52	0.85	2.71	1.28	0.68	2.41	1.02	0.45	2.32	0.74	0.30	1.85
**Hypertension** (Yes vs. No)	**1.88**	**1.05**	**3.36**	1.83	0.97	3.44	**3.27**	**1.57**	**6.83**	**2.68**	**1.23**	**5.82**
**Family history of CVD** (Yes vs. No)	**1.91**	**1.03**	**3.52**	1.33	0.68	2.63	1.70	0.94	3.10	1.61	0.84	3.09
**Ischemic heart disease**^**a**^ (Yes vs. No)	**4.21**	**2.47**	**7.18**	**4.43**	**2.40**	**8.19**	2.13	0.95	4.79	2.27	0.88	5.85
**Atrial fibrillation** (Yes vs. No)	**2.32**	**1.14**	**4.71**	1.24	0.56	2.73	**2.73**	**1.21**	**6.18**	**2.81**	**1.15**	**6.89**
**Other cardiovascular disease**^**b**^ (Yes vs. No)	1.47	0.62	3.50	0.92	0.35	2.41	0.88	0.26	2.95	0.44	0.11	1.80
**Other conditions**^c^												
1–2 vs. 0	1.41	0.81	2.44	1.25	0.69	2.25	1.55	0.85	2.84	1.67	0.86	3.23
3+ vs. 0	**2.50**	**1.09**	**5.75**	**2.81**	**1.15**	**6.89**	0.90	0.29	2.73	0.91	0.28	2.94
**Creatinine** (1 mg/dl increase)	1.91	0.86	4.25	0.80	0.30	2.14	2.11	0.83	5.34	1.25	0.39	3.95

^a^Either angina pectoris, or myocardial infarction, or revascularization procedures; ^b^ Either peripheral vascular disease or valve disease; ^c^ Number of other conditions among stroke, TIA, COPD, liver disease, thyroid disorders, blood disease, gastric disorders, renal disease, Parkinson’s disease, other disease of CNS, and cancer. Fully adjusted ORs are adjusted for all the variables in the table

Table 4 shows the results from fully-adjusted multinomial logistic regression models. (Age-adjusted models are presented in Supplemental Table 1). Given that most of the men with symptomatic DLVD (66%) were HF cases, the factors associated to symptomatic DLVD were those associated to HF: age, ischemic heart disease and comorbid conditions. In men the factors associated with asymptomatic DLVD were age and diabetes. The percentage of HF cases among women with symptomatic DLVD was 57%. In women the factors associated with symptomatic DLVD were age and BMI, whereas the factors associated with asymptomatic DLVD were age and diabetes.

**Table 4:** Association between characteristics of the population and Diastolic Left Ventricular Dysfunction (DLVD) in men and women, results from fully-adjusted multinomial logistic regression. Relative Risk Ratios (RRRs) and 95% Confidence Intervals (CI)

	Men						Women					
	Asymptomatic DLVD			Symptomatic DLVD			Asymptomatic DLVD			Symptomatic DLVD		
	RRR	95% CI		RRR	95% CI		RRR	95% CI		RRR	95% CI	
**Age (1 year increase)**	**1.09**	**1.06**	**1.13**	**1.16**	**1.10**	**1.22**	**1.10**	**1.06**	**1.14**	**1.17**	**1.11**	**1.24**
**Education level**												
Junior High vs. Primary School	1.05	0.67	1.63	0.77	0.38	1.58	0.94	0.63	1.42	1.11	0.56	2.18
> = High vs. Primary School	1.39	0.95	2.03	1.03	0.58	1.82	1.05	0.73	1.53	1.34	0.72	2.51
**Smoking habit (**Ever vs. Never)	0.95	0.68	1.32	1.80	0.96	3.39	1.08	0.77	1.50	1.25	0.71	2.18
**Alcohol consumption** (Yes vs. No)	1.13	0.80	1.59	0.95	0.55	1.63	1.08	0.79	1.48	1.24	0.73	2.10
**Physical activity**												
Yes (occasionally) vs. No	0.80	0.55	1.17	0.76	0.41	1.40	1.03	0.69	1.53	0.57	0.27	1.18
Yes (daily) vs. No	1.00	0.69	1.45	0.65	0.34	1.25	1.04	0.69	1.59	0.61	0.27	1.37
**BMI**												
25–29.9 vs. < 25	1.03	0.74	1.44	1.41	0.80	2.50	1.42	0.91	2.22	**5.36**	**2.50**	**11.49**
30+ vs. < 25	1.31	0.81	2.12	1.90	0.88	4.12	1.14	0.81	1.62	**3.81**	**2.01**	**7.22**
**Dyslipidemia** (Yes vs. No)	0.83	0.60	1.14	**0.52**	**0.30**	**0.90**	0.79	0.58	1.07	0.86	0.51	1.45
**Diabetes (Yes vs. No)**	**1.86**	**1.26**	**2.75**	1.53	0.83	2.83	**1.62**	**1.03**	**2.56**	1.09	0.50	2.37
**Hypertension** (Yes vs. No)	1.16	0.85	1.60	1.47	0.85	2.53	1.05	0.76	1.46	1.58	0.88	2.84
**Family history of CVD** (Yes vs. No)	0.81	0.53	1.22	1.08	0.57	2.03	1.12	0.78	1.61	1.69	0.95	2.99
**Ischemic heart disease**^**a**^ (Yes vs. No)	1.16	0.74	1.82	**3.83**	**2.13**	**6.87**	0.84	0.45	1.57	1.71	0.72	4.04
**Atrial fibrillation** (Yes vs. No)	1.05	0.56	1.95	1.55	0.72	3.34	0.89	0.47	1.69	1.36	0.56	3.33
**Other cardiovascular disease**^**b**^ (Yes vs. No)	0.86	0.43	1.73	0.97	0.39	2.41	1.10	0.56	2.16	1.07	0.38	3.03
**Other conditions**^c^												
1–2 vs. 0	0.78	0.57	1.08	0.95	0.56	1.63	0.86	0.62	1.19	1.66	0.95	2.90
3+ vs. 0	1.20	0.63	2.30	**4.36**	**1.96**	**9.73**	0.68	0.39	1.17	0.29	0.08	1.06
**Creatinine** (1 mg/dl increase)	1.37	0.74	2.54	1.74	0.75	4.03	1.00	0.49	2.02	1.55	0.57	4.21

^a^ Either angina pectoris, or myocardial infarction, or revascularization procedures; ^b^ Either peripheral vascular disease or valve disease; ^c^ Number of other conditions among stroke, TIA, COPD, liver disease, thyroid disorders, blood disease, gastric disorders, renal disease, Parkinson’s disease, other disease of CNS, and cancer; RRRs are adjusted for all the variables

**Fig. 2:**
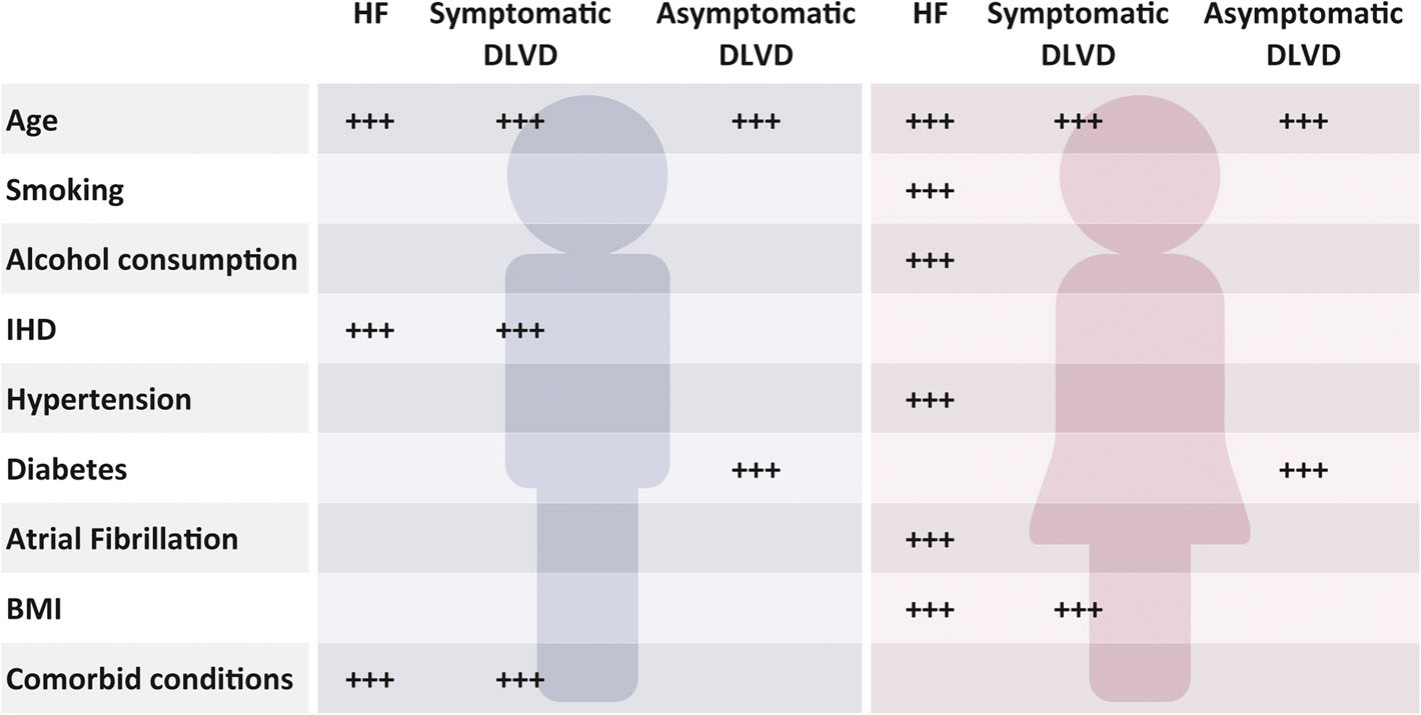
Summary of the results from multivariable analyses

Figure 2 summarizes the main findings of the study.

The weighted multivariate logistic regression showed results comparable to those presented.

## Discussion

In our study population, we found sex differences in the distribution of socio-demographic, lifestyle, and health characteristics and in their association with HF and DVLD. The factors associated with HF in men were age, ischemic heart disease and comorbidities, whereas in women were a combination of lifestyle factors, age, BMI, hypertension, and atrial fibrillation. The association between NT-proBNP and HF was stronger in men than in women. While the factors associated to symptomatic DLVD in men were the same factors associated to HF, in women were age and BMI. This could be related to the HF identification which was based on a mixture of clinical signs and symptoms according to an integrated multi-criteria definition validated at central level by the investigators [14]. In subjects with DVLD and no symptoms, we found sex differences in sociodemographic and clinical characteristics, but we did not observe any difference in relation to the condition. Age and diabetes where the factors associated with asymptomatic DLVD in both men and women.

Attention has recently been paid to the complex issue of sex-specific differences in the impact of cardiovascular risk factors on HF and DLVD [12, 19]. At present, there is no complete understanding of how and why cardiovascular disease presentations differ between sexes. Complex and diverse mechanisms contribute to sex differences in CVD [1, 2, 12]. Several modifiable risk factors for coronary heart disease are similar in men and women. However, some characteristics are stronger predictors of heart disease in women compared to men; for example, hypertension and diabetes are more strongly associated with myocardial infarction in women than men [20].

In our study, age was strongly associated with all the conditions under study in men and in women, confirming previous results [21]. The curve of the relationship between age and HF was steeper in men than in women. However, once all other factors were taken into account, age had a similar association with the condition under study in men and women. Cardiovascular diseases are considered age-related conditions in both sexes. Age-related changes in cardiac and vascular anatomy and physiology, generally called “cardiovascular ageing,” interact over the life course with exposure to traditional risk factors and impact the individual likelihood of developing cardiovascular disease during life [21].

Natriuretic peptides measured in plasma have been shown to be the most effective circulating biomarkers supporting diagnosis, risk stratification and response to treatment in patients with heart failure with reduced ejection fraction. When measured at the same time of the clinical and echocardiographic evaluation of the subjects included in the PREDICTOR cohort, NT-proBNP proved to be higher, irrespective of the presence of HF, in females than in males aged 65+ years. Similar findings in patients/subjects of different ages have been previously reported [22]. The association of logNT-proBNP with HF and diastolic dysfunction was stronger in men than in women. This result contradicts what the Authors of a study in patients of various age with acute HF concluded: NT-proBNP works in females as well as in males when used for differential diagnosis of dyspnoea [23]. The reason for the difference found in community dwelling elderly subjects is not known, but the finding, if confirmed, suggests that NT-proBNP may be a diagnostic of HF in the general population less reliable in females than males. Well known factors influencing the circulating concentrations of natriuretic peptides are age, body mass, and renal function. However, age was similar between males and females in PREDICTOR, and we took account of differences in BMI and in creatinine levels in the analysis. The adoption of diagnostic thresholds for NT-proBNP [24], takes into consideration age, but not sex. The assumption is that the threshold is not influenced by sex, and that the diagnostic performance of the test is similar between sexes, as far as the correct age-based threshold value is used. Our results, if confirmed by other studies, could contribute to the scientific discussion on the validity of this assumption.

In men, ischemic heart disease (IHD) was strongly associated with HF. In women, the risk factors were smoking, alcohol consumption, BMI, hypertension, and atrial fibrillation. In women we did not find evidence of association between IHD and the conditions. Although coronary heart disease is considered to be more related to the male gender, among women it claims more victims than cancer [25]. Cardiovascular diseases (CVDs) differ in men and women in many respects, most notably, the disease manifestations [10, 11]. Although coronary heart disease prevalence is lower in women at any age, this difference is attenuated with advancing age. Men develop cardiovascular diseases earlier than women, even if the overall lifetime risk of CVD is similar in the two sexes [10]. Before menopause, women have a lower risk of IHD than age-matched men [21]. Smoking is a well-known risk factor. However, in our dataset it was associated to HF in women. The absence of association in men does not mean that it is not a risk factor for the male population, but that its association was hidden by the association between IHD and HF in the multivariable analysis.

Although the prevalence of hypertension was similar in men and women, hypertension was associated with HF in the women only. Previous research reported an elevated degree of end-organ damage due to hypertension in women compared to men [26]. Hypertension can contribute to left ventricular and arterial stiffening in a sex-specific way. Sex differences in ventricular diastolic distensibility, vascular stiffness and ventricular/vascular coupling, and skeletal muscle adaptation to HF have been potentially related to a greater rate of diastolic HF in women [26]. The underlying molecular mechanisms include gender differences in calcium handling, the nitric oxide system, and natriuretic peptides [27]. Moreover, oestrogen affects collagen synthesis and degradation and inhibits the renin-angiotensin system. It has been suggested that oestrogens may benefit premenopausal women, and the loss of its protective mechanisms may render the hearts of postmenopausal women vulnerable [28]. Furthermore, although women are more often prescribed antihypertensive drugs than men, blood pressure control is not completely achieved [29]. Differently from the European guidelines, the latest American College of Cardiology/American Heart Association guidelines on arterial hypertension recommend a blood pressure target < 130/80 in all patients with HF; hence, it would be crucial to know if a more intensive pressure target can affect the prognosis to different degrees in the sexes.

Diabetes was associated with asymptomatic DLVD in both men and women, while BMI was associated with symptomatic DLVD in women only. Subjects with pre-diabetes and diabetes are at increased risk of developing HF, either with reduced or preserved ejection fraction. The coexistence of diabetes and HF leads to a higher risk of HF hospitalization, all-cause death, and cardiovascular death. Major causes of HF in patients with diabetes are coronary artery diseases, chronic kidney diseases, hypertension, and myocardial effects of insulin resistance. In patients with severe, diffuse and often silent coronary artery diseases there is an increased risk of cardiovascular events, asymptomatic myocardial dysfunction and HF. Complex pathophysiological mechanisms may be responsible for the development of myocardial dysfunction, even in the absence of coronary artery diseases or hypertension, but the existence of diabetic cardiomyopathy per se has not been confirmed. Association between diabetes and HF with preserved ejection fraction has been reported in hypertensive female patients [30]. As regards BMI, in our population the proportion of normal-weight individuals was higher in women then in men, but so it was the prevalence of obesity. In women, BMI was strongly associated with both HF and DLVD. In the present study however, diabetes was strongly associated with asymptomatic DLVD in both sexes. In multivariable models for DLVD, we found an inverse association between dyslipidaemia and symptomatic DLVD in men. There is not a plausible explanation to this result, that could depend on a statistical artefact. In fact, the association become statistically significant only when ischemic heart diseases are introduced in the model.

The main limitation of this study is its cross-sectional design, which allowed us to analyse the association between risk factors and cardiac dysfunction without knowing the temporal relationship between the two [14]. In addition, another limit is that findings are based on a slightly dated dataset. A common problem in cross-sectional studies is the response rate, as previously reported in our case the response rate was quite low. Moreover, comparing participants and non-participants in our study, high prevalence of women, of elderly, of neurological disability, and of low socio-demographic level was found in non-participants [14]. Hence, there could be problems in generalisability of our results on very old populations, particularly for females. Finally, we based our definition of asymptomatic DLVD on reported symptoms, without considering the impact of functional status. Subjects with low physical activity might report symptoms later than physically active individuals. The strengths of this study include the standardised methodology to measure the risk factors and health status of the study population, the echocardiographic evaluation, and finally yet importantly, that it was a population-based study with a similar number of men and women [19].

## Conclusions

Sex differences seem to influence the diseases in a dissimilar way. Understanding these differences is a key requirement for the early recognition and the early strategies to prevent the disease.

## Supplementary Information

**Additional file 1: Supplemental Figure 1**. Relationship between log NT-proBNP and heart failure (HF), diastolic left ventricular dysfunction (DLVD), and asymptomatic DLVD (ADLVD) in men and women

**Additional file 2: Supplemental Table 1**. Association between characteristics of the population and Diastolic Left Ventricular Dysfunction (DLVD) in men and women, results from multinomial logistic regression adjusted for age. Relative Risk Ratios (RRRs) and 95% Confidence Intervals (CI)

## Data Availability

The data are stored at the Department of Epidemiology-Regional Health Service in Rome, at the S. Giovanni-Addolorata Hospital in Rome, and at the Mario Negri Institute for Pharmacological Research in Milan. For privacy policies of the multicentric PREDICTOR study, data sharing it is not possible.

## Abbreviations

ADLVD: Asymptomatic diastolic left ventricular dysfunction
ARB: Angiotensin II Receptor Blockers
BMI: Body mass index
CI: Confidence interval
CVD: Cardiovascular disease
DLVD: Diastolic left ventricular dysfunction
HF: Heart failure
IHD: Ischemic heart disease
OR: Odds ratios
SDLVD: Symptomatic diastolic left ventricular dysfunction

## References

[1] Yancy CW, Jessup M, Bozkurt B, Butler J, Casey DE, Colvin MM (2017). 2017 ACC/AHA/HFSA focused update of the 2013 ACCF/AHA guideline for the Management of Heart Failure: a report of the American College of Cardiology/American Heart Association task force on clinical practice guidelines and the heart failure Society of Amer. Circulation.

[2] Redfield MM, Jacobsen SJ, Burnett JC, Mahoney DW, Bailey KR, Rodeheffer RJ (2003). Burden of Systolic and Diastolic Ventricular Dysfunction in the Community. JAMA.

[3] Chioncel O, Lainscak M, Seferovic PM, Anker SD, Crespo-Leiro MG, Harjola VP, et al. Epidemiology and one-year outcomes in patients with chronic heart failure and preserved, mid-range and reduced ejection fraction: an analysis of the ESC Heart Failure Long-Term Registry Methods and results. Eur J Heart Fail. 2017:1–12. 10.1002/ejhf.813. 10.1002/ejhf.813 28386917

[4] Krum H, Abraham WT (2009). Heart failure. Lancet.

[5] Arnold AP, Cassis LA, Eghbali M, Reue K, Sandberg K (2017). Sex hormones and sex chromosomes cause sex differences in the development of cardiovascular diseases. Arterioscler Thromb Vasc Biol.

[6] Lo RC, Bensley RP, Dahlberg SE, Matyal R, Hamdan AD, Wyers M (2014). Presentation, treatment, and outcome differences between men and women undergoing revascularization or amputation for lower extremity peripheral arterial disease. J Vasc Surg.

[7] Maric-Bilkan C (2017). Sex differences in micro- and macro-vascular complications of diabetes mellitus. Clin Sci.

[8] Matthan NR, Zhu L, Pencina M, D'Agostino RB, Schaefer EJ, Lichtenstein AH (2013). Sex-specific differences in the predictive value of cholesterol homeostasis markers and 10-year cardiovascular disease event rate in Framingham offspring study participants. J Am Heart Assoc.

[9] McGregor AJ, Frank Peacock W, Marie Chang A, Safdar B, Diercks D (2014). Sex- and gender-specific research priorities for the emergency management of heart failure and acute arrhythmia: proceedings from the 2014 academic emergency medicine consensus conference cardiovascular research workgroup. Acad Emerg Med.

[10] Leening MJG, Ferket BS, Steyerberg EW, Safdar B, Diercks D (2014). Sex differences in lifetime risk and first manifestation of cardiovascular disease: prospective population based cohort study. Bmj.

[11] Mosca L, Barrett-Connor E, Kass Wenger N (2011). Sex/gender differences in cardiovascular disease prevention: what a difference a decade makes. Circulation.

[12] Mauvais-Jarvis F, Bairey Merz N, Barnes PJ, Brinton RD, Carrero JJ, DeMeo DL (2020). Sex and gender: modifiers of health, disease, and medicine. Lancet..

[13] Ponikowski P, Voors AA, Anker SD, Bueno H, Cleland JGF, Coats AJS, ESC Scientific Document Group (2016). 2016 ESC Guidelines for the diagnosis and treatment of acute and chronic heart failure: The Task Force for the diagnosis and treatment of acute and chronic heart failure of the European Society of Cardiology (ESC) Developed with the special contribution of the Heart Failure Association (HFA) of the ESC. Eur Heart J.

[14] Mureddu GF, Agabiti N, Rizzello V, Forastiere F, Latini R, Cesaroni G (2012). Prevalence of preclinical and clinical heart failure in the elderly. A population-based study in Central Italy. Eur J Heart Fail.

[15] Masson S, Latini R, Mureddu GF, Agabiti N, Miceli M, Cesaroni G (2013). High-sensitivity cardiac troponin T for detection of subtle abnormalities of cardiac phenotype in a general population of elderly individuals. J Intern Med.

[16] Eisen EA, Agalliu I, Thurston SW, Coull BA, Checkoway H (2004). Smoothing in occupational cohort studies: an illustration based on penalised splines. Occup Environ Med.

[17] Mansournia MA, Altman DG (2016). Inverse probability weighting. BMJ..

[18] Narduzzi S, Golini MN, Porta D, Stafoggia M, Forastiere F (2014). Inverse probability weighting (IPW) for evaluating and "correcting" selection bias. Epidemiol Prev.

[19] Pucci G, Alcidi R, Tap L, Battista F, Mattace-Raso F, Schillaci G (2017). Sex- and gender-related prevalence, cardiovascular risk and therapeutic approach in metabolic syndrome: a review of the literature. Pharmacol Res.

[20] Meercuro G, Deidda M, Piras A, Dessalvi CC, Maffei S, Rosano GM (2010). Gender determinants of cardiovascular risk factors and diseases. J Cardiovasc Med.

[21] Merz AA, Cheng S (2016). Sex differences in cardiovascular ageing. Heart.

[22] Sobhani K, Nieves Castro DK, Fu Q, Gottlieb RA, Van Eyk JE, Noel Bairey Merz C (2018). Sex differences in ischemic heart disease and heart failure biomarkers. Biol Sex Differ.

[23] Krauser DG, Chen AA, Tung R, Anwaruddin S, Baggish AL, Januzzi JL (2006). Neither race nor gender influences the usefulness of amino-terminal pro-brain natriuretic peptide testing in dyspneic subjects: a ProBNP investigation of dyspnea in the emergency department (PRIDE) substudy. J Card Fail.

[24] Januzzi JL, Chen-Tournoux AA, Christenson RH, Doros G, Hollander JE, Levy PD, ICON-RELOADED Investigators (2018). N-Terminal Pro-B-Type NatriureticPeptide in the Emergency Department: The ICON-RELOADED Study. J Am Coll Cardiol.

[25] Collaborators GBD 2013 Mortality and (2015). Global, regional, and national age–sex specific all-cause and cause-specific mortality for 240 causes of death, 1990–2013: a systematic analysis for the global burden of disease study 2013. Lancet.

[26] Crislip GR, Sullivan JC (2016). T-cell involvement in sex differences in blood pressure control. Clin Sci.

[27] Gori M, Lam CSP, Gupta DK, Santos AB, Cheng S, Shah AM (2014). Sex-specific cardiovascular structure and function in heart failure with preserved ejection fraction methods and results. Eur J Heart Fail.

[28] Hayward CS, Kelly RP, Collins P (2000). The roles of gender, the menopause and hormone replacement on cardiovascular function. Cardiovasc Res.

[29] Shah T, Palaskas N, Ahmed A. An update on gender disparities in coronary heart disease care. Curr Atheroscler Rep. 2016;18. 10.1007/s11883-016-0574-5. 10.1007/s11883-016-0574-5 27029220

[30] Grant PJ, Cosentino F (2019). The 2019 ESC guidelines on diabetes, pre-diabetes, and cardiovascular diseases developed in collaboration with the EASD: new features and the ‘ten commandments’ of the 2019 guidelines are discussed by professor Peter J. Grant and professor Francesco Cosentino, the task force chairmen. Eur Heart J.

