# Supplementary figures and images for: Sex differences in factors associated with heart failure and diastolic left ventricular dysfunction: a cross-sectional population-based study
Source: BMC Public Health. 2021 Feb 27;21:415. doi: 10.1186/s12889-021-10442-3 (PMC7912519; doi:10.1186/s12889-021-10442-3)

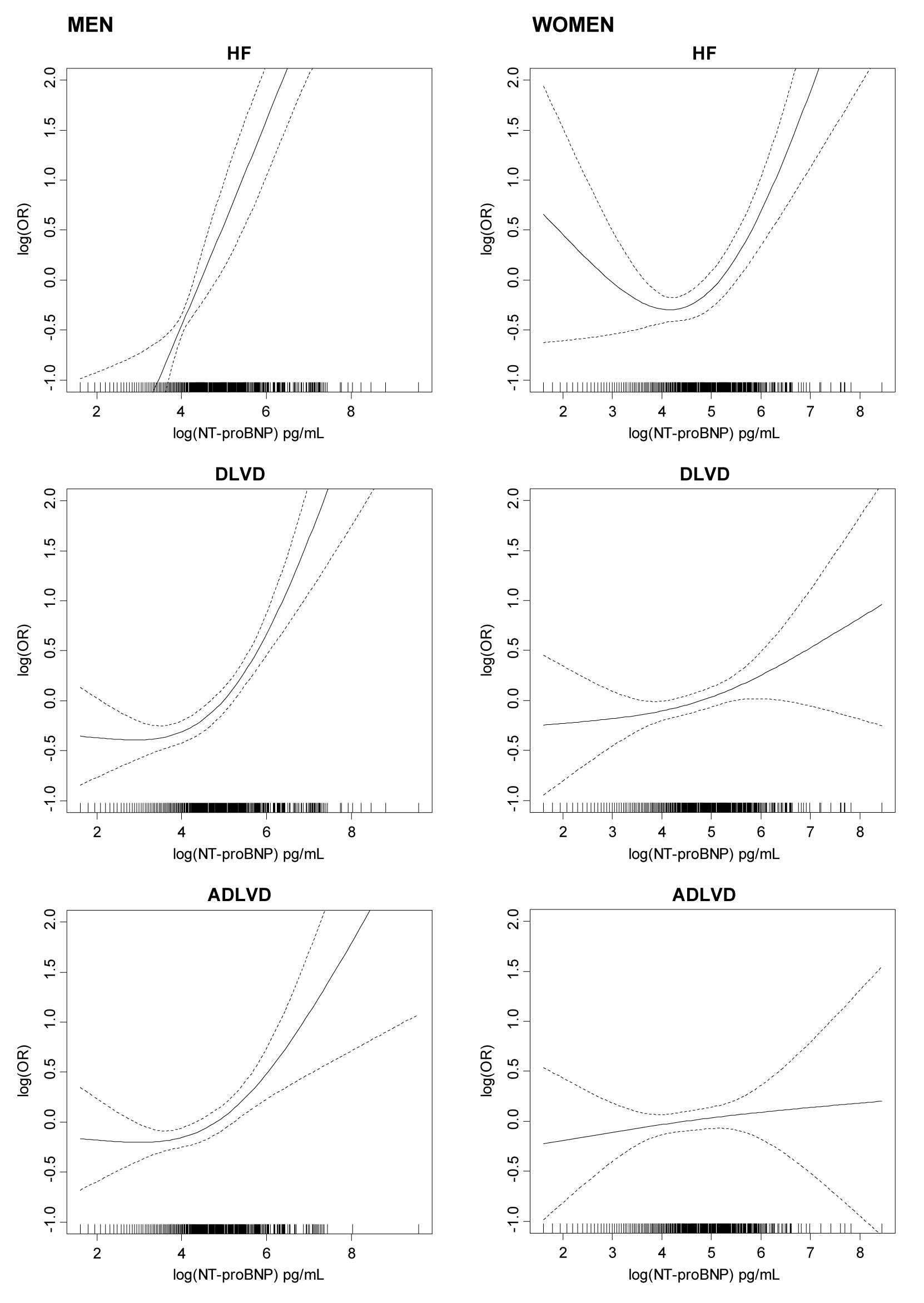

Supplement: Supplementary file 1 — Additional file 1: Supplemental Figure 1. Relationship between log NT-proBNP and heart failure (HF), diastolic left ventricular dysfunction (DLVD), and asymptomatic DLVD (ADLVD) in men and women [file 12889_2021_10442_MOESM1_ESM.tif]
