# Supplementary material for: Sex differences in factors associated with heart failure and diastolic left ventricular dysfunction: a cross-sectional population-based study
Source: BMC Public Health. 2021 Feb 27;21:415. doi: 10.1186/s12889-021-10442-3 (PMC7912519; doi:10.1186/s12889-021-10442-3)
Supplement: Supplementary file 2 — Additional file 2: Supplemental Table 1. Association between characteristics of the population and Diastolic Left Ventricular Dysfunction (DLVD) in men and women, results from multinomial logistic regression adjusted for age. Relative Risk Ratios (RRRs) and 95% Confidence Intervals (CI) [file 12889_2021_10442_MOESM2_ESM.docx]

| **Supplemental Table 1. Association between characteristics of the population and Diastolic Left Ventricular Dysfunction (DLVD) in men and women, results from multinomial logistic regression adjusted for age. Relative Risk Ratios (RRRs) and 95% Confidence Intervals (CI)** | | | | | | | | | | | | |
| --- | --- | --- | --- | --- | --- | --- | --- | --- | --- | --- | --- | --- |
|  | **Men** | | | | | | **Women** | | | | | |
|  | **Asymptomatic DLVD** | | | **Symptomatic DLVD** | | | **Asymptomatic DLVD** | | | **Symptomatic DLVD** | | |
|  | **RRR** | **95% CI** | | **RRR** | **95% CI** | | **RRR** | **95% CI** | | **RRR** | **95% CI** | |
| **Age (1 year increase)** | **1.09** | **1.06** | **1.13** | **1.18** | **1.13** | **1.24** | **1.09** | **1.06** | **1.13** | **1.15** | **1.09** | **1.20** |
| **Education level** |  |  |  |  |  |  |  |  |  |  |  |  |
| Junior High vs. Primary School | 1.02 | 0.66 | 1.56 | 0.70 | 0.36 | 1.38 | 0.90 | 0.61 | 1.34 | 0.93 | 0.49 | 1.74 |
| >= High vs. Primary School | 1.26 | 0.88 | 1.80 | 0.87 | 0.51 | 1.46 | 0.95 | 0.67 | 1.33 | 0.88 | 0.50 | 1.54 |
| **Smoking habit (**Ever vs. Never) | 0.96 | 0.70 | 1.31 | **2.01** | **1.12** | **3.61** | 1.03 | 0.75 | 1.41 | 1.09 | 0.65 | 1.83 |
| **Alcohol consumption** (Yes vs. No) | 1.07 | 0.77 | 1.50 | 0.85 | 0.52 | 1.41 | 1.05 | 0.78 | 1.42 | 1.00 | 0.61 | 1.63 |
| **Physical activity** |  |  |  |  |  |  |  |  |  |  |  |  |
| Yes (occasionally) vs. No | 0.82 | 0.57 | 1.19 | 0.82 | 0.46 | 1.46 | 1.04 | 0.71 | 1.52 | 0.61 | 0.30 | 1.23 |
| Yes (daily) vs. No | 0.95 | 0.66 | 1.36 | 0.63 | 0.34 | 1.16 | 1.02 | 0.68 | 1.53 | 0.63 | 0.30 | 1.30 |
| **BMI** |  |  |  |  |  |  |  |  |  |  |  |  |
| 25-29.9 vs. <25 | 1.06 | 0.76 | 1.47 | 1.46 | 0.85 | 2.48 | 1.16 | 0.83 | 1.63 | **3.96** | **2.15** | **7.28** |
| 30+ vs. <25 | 1.39 | 0.88 | 2.20 | **2.06** | **1.01** | **4.24** | 1.50 | 0.98 | 2.30 | **5.32** | **2.62** | **10.78** |
| **Dyslipidemia** (Yes vs. No) | 0.95 | 0.70 | 1.29 | 0.80 | 0.49 | 1.32 | 0.80 | 0.59 | 1.08 | 0.89 | 0.55 | 1.45 |
| **Diabetes (Yes vs. No)** | **1.87** | **1.29** | **2.71** | **1.77** | **1.00** | **3.13** | 1.54 | 0.99 | 2.37 | 1.43 | 0.73 | 2.83 |
| **Hypertension** (Yes vs. No) | 1.22 | 0.91 | 1.65 | **1.69** | **1.03** | **2.77** | 1.11 | 0.81 | 1.50 | **2.13** | **1.23** | **3.69** |
| **Family history of CVD** (Yes vs. No) | 0.84 | 0.57 | 1.26 | 1.47 | 0.83 | 2.60 | 1.07 | 0.75 | 1.52 | **1.72** | **1.02** | **2.91** |
| **Ischemic heart disease^a^** (Yes vs. No) | 1.16 | 0.77 | 1.75 | **3.95** | **2.36** | **6.60** | 0.91 | 0.50 | 1.63 | 1.62 | 0.74 | 3.53 |
| **Atrial fibrillation** (Yes vs. No) | 0.98 | 0.54 | 1.78 | **2.55** | **1.28** | **5.07** | 0.90 | 0.48 | 1.67 | 1.72 | 0.76 | 3.88 |
| **Other cardiovascular disease^b^** (Yes vs. No) | 0.84 | 0.44 | 1.62 | 1.52 | 0.67 | 3.42 | 0.98 | 0.52 | 1.86 | 1.40 | 0.57 | 3.44 |
| **Other conditions^c^** |  |  |  |  |  |  |  |  |  |  |  |  |
| 1-2 vs. 0 | 0.81 | 0.60 | 1.11 | 1.12 | 0.68 | 1.85 | 0.87 | 0.64 | 1.20 | 1.58 | 0.94 | 2.65 |
| 3+ vs. 0 | 1.19 | 0.63 | 2.24 | **3.92** | **1.84** | **8.36** | 0.67 | 0.40 | 1.12 | 0.46 | 0.15 | 1.39 |
| **Creatinine** (1mg/dl increase) | 1.47 | 0.81 | 2.66 | **2.30** | **1.04** | **5.05** | 0.98 | 0.50 | 1.92 | 1.80 | 0.74 | 4.35 |
| ^a^ Either angina pectoris, or myocardial infarction, or revascularization procedures | | | | | | |  |  |  |  |  |  |
| ^b^ Either peripheral vascular disease or valve disease | | |  |  |  |  |  |  |  |  |  |  |
| ^c^ Number of other conditions among stroke, TIA, COPD, liver disease, thyroid disorders, blood disease, gastric disorders, renal disease, Parkinson's disease, other disease of CNS, and cancer | | | | | | | | | | | | |
